# In and out of the loop: external and internal modulation of the olivo-cerebellar loop

**DOI:** 10.3389/fncir.2013.00073

**Published:** 2013-04-19

**Authors:** Avraham M. Libster, Yosef Yarom

**Affiliations:** ^1^Department of Neurobiology, Life Science Institute, Hebrew UniversityJerusalem, Israel; ^2^Edmund and Lily Safra Center for Brain Sciences, Hebrew UniversityJerusalem, Israel

**Keywords:** cerebellum, neuromodulation, olivo-cerebellar loop, aminergic modulation, inferior olive, cerebellar nuclei, cerebellar cortex

## Abstract

Cerebellar anatomy is known for its crystal like structure, where neurons and connections are precisely and repeatedly organized with minor variations across the Cerebellar Cortex. The olivo-cerebellar loop, denoting the connections between the Cerebellar cortex, Inferior Olive and Cerebellar Nuclei (CN), is also modularly organized to form what is known as the cerebellar module. In contrast to the relatively organized and static anatomy, the cerebellum is innervated by a wide variety of neuromodulator carrying axons that are heterogeneously distributed along the olivo-cerebellar loop, providing heterogeneity to the static structure. In this manuscript we review modulatory processes in the olivo-cerebellar loop. We start by discussing the relationship between neuromodulators and the animal behavioral states. This is followed with an overview of the cerebellar neuromodulatory signals and a short discussion of why and when the cerebellar activity should be modulated. We then devote a section for three types of neurons where we briefly review its properties and propose possible neuromodulation scenarios.

## Introduction

The close relationships between the psychiatric state and the motor system is beautifully demonstrated in a clinical report describing a post-traumatic disorder case where exposure to loud sound lead to tremors lasting from several minutes to several days (Walters and Hening, 1992). The observed reaction to loud sound is a classic symptom of Psychogenic Tremor (PT), a movement disorder classified as Psychogenic Motor Disorder (PMD) (Jankovic et al., 2006). PMD, as its name suggests, is a movement disorder having a psychological origin (Association, 2000) and it clearly demonstrate that the properties of the motor control system can be altered on a transition to a different behavioral state.

The shifts between different behavioral states are commonly observed in animal behavior (Irwin, 1968). The shifts, which can be triggered by either internal or external stimuli, can be accompanied by changes in motor activity. Switching between the different states allows the animal to cope with changes that happened or about to happen in the external world. It is of no surprise that each behavioral state is accompanied by a distinct global brain activity manifested over different brain areas. A variety of physiological measurements, EEG (Lindsley, 1952), LFP (Gervasoni et al., 2004), and single unit activity (Abeles et al., 1995; Fanselow et al., 2001; Steriade et al., 2001), were used to characterize a state related brain activity [reviewed in Lee and Dan (2012)].

Changing global brain activity doesn't seem to be a cascading event but rather a simultaneous modification in activity of many parts of the CNS. The coordinated modification is regulated by a set of subcortical structures, each composed of neurons containing aminergic substances (Graeff et al., 1996; Everitt and Robbins, 1997; Taheri et al., 2002; Berridge and Waterhouse, 2003; Burgess, 2010). These aminergic substances, operates as neuromodulators, binding to specific, usually metabotropic membrane receptors. Upon binding they affect both, the cells intrinsic properties and the properties of the synaptic inputs. Neuromodulators can be co-released with neurotransmitters, such as glutamate (Trudeau, 2004), either at or in the vicinity of the synaptic site. Alternatively neuromodulators can have a global effect via what is known as volume transmission. A neuromodulator is said to be volume transmitted when it release sites and the matching receptors, in the target area, are relatively far from each other (Agnati et al., 2006).

Measuring the activity of neurons in these subcortical areas shows correlation between their activity and the animal behavioral state, i.e., both serotonergic neurons in the Dorsal Reticular Nucleus (DRN) and noradrenergic neurons in the Locus Coeruleus (LC) increase their rate of activation as the animal shifts from REM sleep through quite wakefulness to attentive behavior (Hobson et al., 1975; Trulson and Jacobs, 1979; Veasey et al., 1997; Jacobs et al., 2002). The role of aminergic neurons has been recently supported by experiments using optogenetic tools, showing that specific alteration of the activity in these areas affected the animal sleep-awake cycle and motor behavior (Carter et al., 2010; McGregor and Siegel, 2010), social behavior (Chaudhury et al., 2013) and attention (Narayanan et al., 2012).

Within a behavioral state the neuromodulatory system operates in two release modes: tonic and phasic. While tonic release, which lasts throughout the behavioral state, regulates non-specific aspects, the phasic release is activated by specific stimuli or during specific task. For example dopaminergic neurons encode the predicted reward of stimuli (Schultz et al., 1997; Hollerman and Schultz, 1998), neurons in the LC have different responses to target or distractor stimuli in visual discrimination tasks (Aston-Jones et al., 1999) and serotonergic neurons in the DRN exhibit stimulus related (Ranade and Mainen, 2009) and specific motor activity (Veasey et al., 1995; Jacobs and Fornal, 1997) related firing.

## Extrinsic and intrinsic modulation

Neuromodulation in the CNS can be divided to extrinsic and intrinsic systems. The subcortical structures described in the previous section are extrinsic, as they are located outside the modulated target structure and operate almost independently of the target structure's activity. In intrinsic system the source of the modulating substance is within the structure and its release is almost entirely dependent on the activity of the local neural circuit. Another distinction between the systems is the possibility of activation of only subset of the secretory cells thus providing the intrinsic neuromodulatory system with a better spatial resolution.

In the case of the cerebellum, some of the extrinsic neuromodulators are: 5-HT, Dopamine, Ach, NE, Orexin and Histamine. The release pattern of these neuromodulators is relatively independent of the activity of the olivo-cerebellar loop [i.e., dopamine, (Rogers et al., 2011)]. Intrinsic neuromodulators, such as CRF [reviewed by Ito (2009)], Endocannabinoids (Safo et al., 2006), NO (Shibuki and Okada, 1991; Saxon and Beitz, 1996) and glutamate (Kano et al., 2008), are mostly produced and released within, and under the control of the olivo-cerebellar loop.

## The cerebellum in different behavioral states

**Figure 1:**
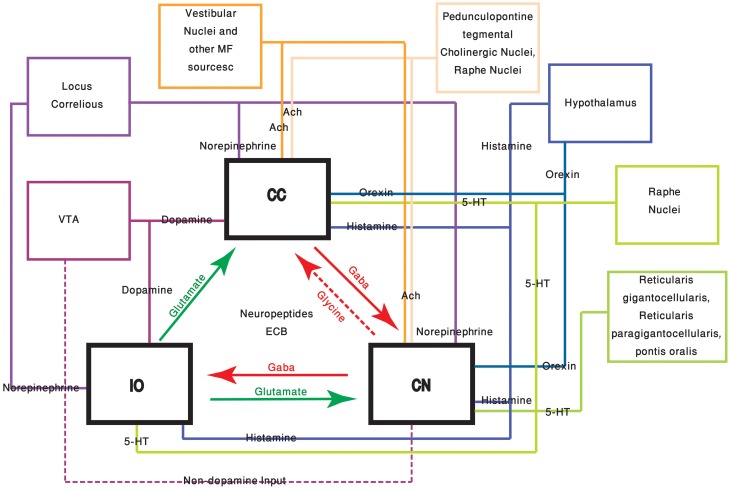
**The olivo-cerebellar and neuromodulation sources.** The different parts of the olivo-cerebellar loop, Cerebellar Cortex (CC), Inferior Olive (IO), and Cerebellar Nuclei (CN) are schematically drawn. Excitatory and inhibitory connections between them are marked in green and red arrows respectively. Each box represents an external source of neuromodulation to the loop. The represented neuromodulators are: 5-HT—marked in light green. Notice the diagram doesn't include the contribution of 5-HT from serotonerigic neurons located in the precerebellar areas. Orexin and histamine—marked, respectively, in light and dark blue. Both are secreted from cells located in different areas in the hypothalamus. Ach—marked in light and dark orange. The two main sources are precerebellar regions and nuclei in the reticular formation. Note that cholinergic input to the IO is not represented in the diagram. Norepinephrine—marked in dark purple. Dopamine—marked in light purple. Dashed line represents input from the VTA to the CN which is not dopaminergic. Autocrine signaling of dopamine in PCs (Kim et al., 2009) is not represented in the scheme.

The neuromodulators of the cerebellum are well documented. Numerous subcortical areas such as the hypothalamus (Dietrichs and Haines, 1989), Ventral Tegmental Area (VTA) (Ikai et al., 1992, 1994), DRN (Pierce et al., 1977; Mendlin et al., 1996) and LC (Somana and Walberg, 1979) provide various modulatory agents (Figure 1) and their effects on different parts of the olivo-cerebellar loop, both *in vivo* and *in vitro* were documented (Schweighofer et al., 2004). Yet, the role of the cerebellum in different behavioral states was largely ignored.

**Figure 2:**
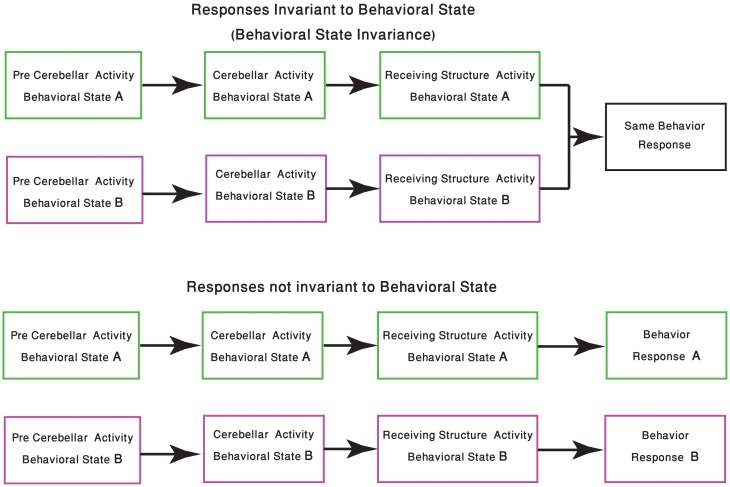
**Change in response due the behavioral state shift.** The schematic diagram represents the two possible outcomes of cerebellar processing which is dependent on the animal behavioral state. In the upper panel the same sensory-motor information is presented to the cerebellar system, tough due to changes in precerebellar regions, the representation of this sensory-motor information might differ. In both behavioral states the cerebellum produces an output, received by other structures in the CNS, resulting in the animal having the same behavioral response. The lower panel describes a scenario in which the behavioral response should be different. Neuromodulation processes, occurring in different states, should be able to select the behavioral responses which are kept invariant.

One of the few studies on cerebellar function and its relation to behavioral state, recently published by Wu et al., demonstrated that the timing activity in the cerebellum is awareness independent. It concludes that coding of the sensory stimuli timing is largely independent of “… attentional, top-down or cognitive control mechanisms” (Wu et al., 2011). Although it might imply that cerebellar function is independent of the behavioral state, we argue that in order to preserve the cerebellar “timing function,” and given that the cerebellar circuitry is modified upon the shift in the behavioral state, one have to change the “coding of time.” In generalizing this idea, we argue that given a global change of brain activity, the input to the cerebellum and the response to cerebellar output are bound to change. Therefore, cerebellar activity most be modified in order to either ensure that the response is independent of the behavioral state or to provide a response that fit the behavioral requirements of the new state (Figure 2).

In the following sections we will review the effects of neuromodulators on one cell type from each of the constituents of the olivo-cerebellar loop: the Cerebellar Nuclei (CN) neuron, the Purkinje Cell (PC) and olivary neurons. For each cell type, we will describe one of its many observed electrophysiological phenomena and speculate on possible modulation scenarios.

## The CN neurons

### The biophysical properties of CN neurons

**Figure 3:**
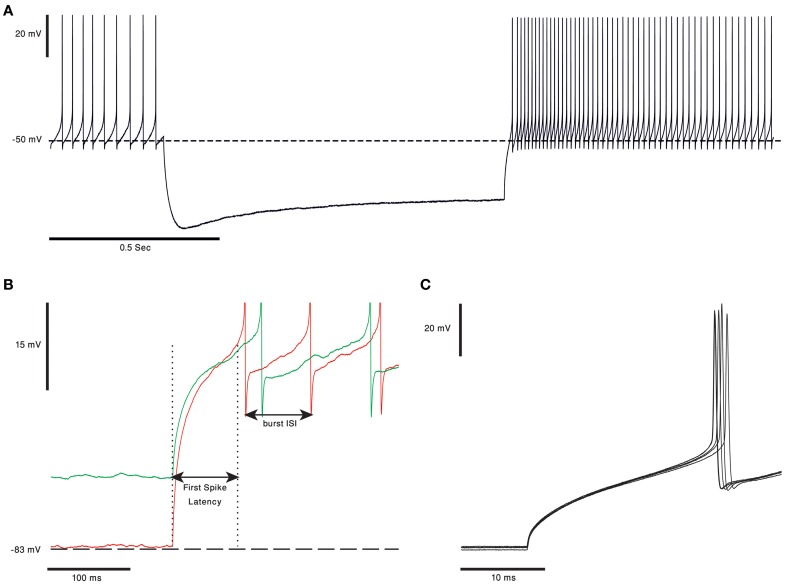
**Rebound burst in CN neurons. (A)** Voltage trace from *in vitro* recording of a bPN. In response to 1 s of hyperpolarization the cell responds with a long rebound burst response, as evident in the increase in firing rate. Notice the voltage “sag” clearly visible during the hyperpolarization of the membrane voltage, probably due to activation of HCN channels (the voltage traces are courtesy of Dr. Uusisaari). **(B)** Rebound burst in response to different hyperpolarization levels. The first spike latency was shorter and burst frequency was higher when the membrane voltage was more hyperpolarized (red). Spikes were cut for better visualization. **(C)** The first spikes in the rebound burst from five repeats of the protocol, from panel **(A)**, done on the same cell. The latency to the first spike has a clear and noticeable small jitter.

One of the ongoing debates in the field of cerebellar research is whether the output of the cerebellar cortex is conveyed via the rebound burst occurring in the CN neurons (Alviña et al., 2008; Boehme et al., 2011). Rebound burst (see Figure 3A) is a high frequency spikes burst triggered by a prolonged period of hyperpolarization (Tadayonnejad et al., 2010). The rebound response in CN neurons is mediated by the activation of T-type calcium channels (Cav3.x) and HCN channels (Molineux et al., 2006, 2008; Alviña et al., 2009; Engbers et al., 2011). The expression of either Cav3.1, or Cav 3.3, governs the number of spikes in the rebound burst and their inter-spike-intervals (see Figure 3B). The activation of these channels, expressed in the soma and non-uniformly distributed along the dendrites (Gauck et al., 2001; McKay et al., 2006), trigger either “strong” or “weak” bursts reported *in vitro* (Molineux et al., 2006). The HCN channels generate a non-specific, slowly inactivated cationic current (h-current). This current, which is activated by membrane hyperpolarization (Wahl-Schott and Biel, 2009), contributes to the rebound response by increasing the depolarization at the end of a hyperpolarizing period. The depolarization will act to increase the intra-burst firing rate and decrease the variance of the latency to the first spike (Engbers et al., 2011) (Figure 3C). The three types of HCN channels found in the CN neurons are HCN1, HCN2 and HCN 4. The HCN variants are spatially segregated: HCN2 is located proximally whereas HCN4 is found mainly at the distal dendrites (Santoro et al., 2000; Notomi and Shigemoto, 2004). Out of the three HCN isoforms, HCN2, and HCN4 are more susceptible to regulation by cAMP levels. An increase in intracellular cAMP concentration causes a rightward shift of the HCN activation curve and induces faster opening kinetics (Wahl-Schott and Biel, 2009).

### Neuromodulation of CN neurons

Table 1 summarizes some of the current knowledge on the neuromodulators operating within the CN. Here we describe presumable modulation strategies that can change the output of the CN by altering either the “rebound response” or modulating the CN inputs.

**Table 1:** **Neuromodulators of CN**.

**Neuromodulator**	**Source**	**Receptors**	**Known effects**
5-HT	GiC, PnO (Bishop and Ho, 1985).	5-HT1B (might be expressed by PCs axons) 5-HT1C,5-HT2A, 5-HT2B (Might be expressed only in IN) 5-HT3 (low levels), 5-HT5A (Choi and Maroteaux, 1996; Kia et al., 1996; Sari et al., 1999; Geurts et al., 2002).	*In vitro*
			Attenuates the HCN current and decreases the amplitude of IPSCs by a presynaptic mechanism (Saitow et al., 2009). Increases the firing rate and reduces the response to glutamate via a postsynaptic mechanism (Gardette et al., 1987).
			*In vivo*
			5-HT1A and 5-HT2 agonists induce a decrease in firing rate and 5-HT5A agonist increase firing rate (Di Mauro et al., 2003). In other studies only decreases in firing rate and response to glutamate, were documented (Kitzman and Bishop, 1997). Neurons excited by 5-HT were located at cerebellar nuclei projecting to the thalamus and cortex, whereas the nuclei projecting to peripheral motor centers reduced their firing rate when levels of 5-HT increased (Di Mauro et al., 2003).
NE	LC (Hokfelt and Fuxe, 1969; Somana and Walberg, 1978).		*In vivo*
			Direct application of NE decreases the firing rate of neurons in all nuclei (Di Mauro et al., 2003).
			Decreases response to application of GABA in the FN and Posterior IN while increasing it in the anterior IN. The LN has mixed responses (Di Mauro et al., 2012).
Ach	Vestibular nuclei (non-beaded fibers) PTg, GiC and Raphe nuclei (beaded fibers creating a dense network) (Jaarsma et al., 1997).		
Dopamine	But source of dopamine is unknown as nuclei is innervated by non-dopaminergic neurons from the VTA (Ikai et al., 1992).	DAT presence is demonstrated (Delis et al., 2004, 2008).	
Histamine	TMN (Haas and Panula, 2003).	H1, H2 (Qin et al., 2011) and H3 (mRNA in FN and IN) (Pillot et al., 2002).	*In vitro*
			Increases firing rate of neurons in all of the CN, probably through H2 activation (Shen et al., 2002; Tang et al., 2008; Qin et al., 2011).
Orexin	PeFLH (Peyron et al., 1998).	OX1R, OX2R (Hervieu et al., 2001; Cluderay et al., 2002).	*In vitro*
			Increases firing rate of neurons in the IN probably through OX2R activation (Yu et al., 2010).

The most straightforward modulation of rebound response is to change the kinetic of either the calcium current, the h-current or both. Modulation of the t-type or HCN channel kinetics can either change the frequency of spikes in the burst or the latency to the first spike (Figure 3A inset) (Engbers et al., 2011). While changing the time of the first spike is bound to change the “time representation,” changing the frequency of the burst will alter the intensity of the CN output. The later may reflect the need to adapt to the new behavioral state.

HCN channels, as mentioned above, are regulated by the intracellular levels of cAMP and cGMP. Since many neuromodulatory pathways use cAMP and cGMP as second messengers, the modulation of HCN channels during a shift in behavioral state is likely to occur. Therefore, we will consider possible interesting scenarios of HCN modulation.

One of the intriguing neuromodulation scenarios is the differential effect on specific input. Neurons assign “value” to the different inputs carried by afferents from diverse pathways. This “value” is determined by either the location of the input or its relative strength. Differential neuromodulation can occur if the neuromodulators receptors are non-homogeneously distributed [This scenario, among many others, is discussed in (Dayan, 2012)].

The big Projection Neurons (bPNs) of the CN are suited for differential neuromodulation. A typical bPN receives excitatory input from the Mossy Fibers (MF) and Climbing Fibers (CF) collaterals and inhibitory input from PCs and local interneurons. Studies have shown that, at least in the Dentate Nucleus (DN), PCs synapses are located at the soma with a decreasing gradient along the dendrites. The MF excitatory input, on the other hand, is mostly located on the distal parts of the dendritic tree, as oppose to the CF input that is found on proximal dendrites (Chan-Palay, 1977; Uusisaari and Knöpfel, 2011). Furthermore, a non-homogeneous distribution of HCN channel has been reported (Santoro et al., 2000; Notomi and Shigemoto, 2004; Wilson and Garthwaite, 2010) as well as location specific innervations of neuromodulators [i.e., the cholinergic system has synaptic junctions close to the dendrites (Jaarsma et al., 1997)]. As a result of this high degree of non-uniformity, neuromodulation can be highly specific. For example, modulation of HCN channels located at the distal part of the dendrites will affect the input from the MF while modulation of the more proximal parts of the dendrite can affect the CF input. This modulation strategy enables the bPN to selectively augment or attenuate EPSPs of different input sources. This “input” targeted modulation, can be viewed as a way of differentially changing the “sensitivity” of a bPN to inputs from the olivo-cerebellar loop or external input from precerebellar regions. Interestingly, this effect might also be achieved by modulating T-type calcium channels. The T-type channels are expressed in distal parts of the dendrite (Gauck et al., 2001) and might play a role in amplifying the excitatory input as seen in other parts of the CNS (i.e., Urban et al., 1998).

What are the advantages of having a differential modulation of rebound burst and excitatory input? We may answer it by assuming a different “expected” bPN output during different input regimes. When most of the inputs are inhibitory, prolonged hyperpolarization of the bPN membrane potential will enable the rebound burst mechanism. The rebound burst can then be considered as the “expected” output. In this case modulation of the rebound burst properties would have it largest effect on the information, conveyed by the bPN to the rest of CNS. On the other hand when the bPN receives prolonged excitatory drive, inhibition will modulate the timing and intensity of bPN response (Holdefer et al., 2005). In the “excitatory” input regime, modulation of EPSPs and spiking probability will have a larger effect on the information, conveyed by the bPN to the rest of CNS, then changing the properties of the rebound burst.

## The Purkinje cells

### The biophysical properties of purkine cells

The properties of cerebellar PC have been extensively studied both *in vitro* and *in vivo* in anaesthetized and awake animals. It is commonly accepted that these unique neurons are endowed with a variety of ionic channels that provide a large repertoire of electrical activity (Llinas and Sugimori, 1980a,b; Williams et al., 2002). It is beyond the scope of this manuscript to review the vast literature describing the electrophysiological properties of PC and therefore we will limit our description to few of these properties. The three main ionic currents that control the firing of PC are Na, Ca and h-current. To this short list one should add a variety of potassium currents that are either voltage dependent, calcium dependent or both. While the first three currents control the excitability of the neurons, the potassium currents have a prominent role in shaping the frequency and pattern of activity (Womack and Khodakhah, 2002, 2004). The kinetics of most, if not all, of these ionic currents can be modified by neuromodulators, resulting in a profound change in the electrical behavior.

**Figure 4:**
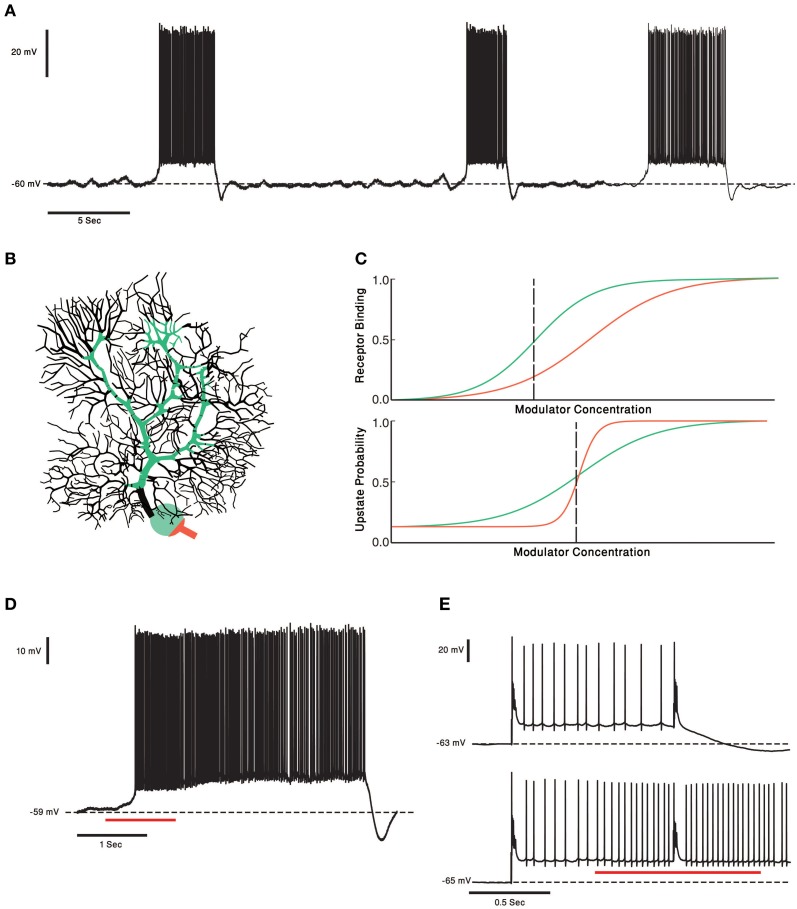
**PCs and two Receptors modulation. (A)** Voltage trace from *in vitro* recording of a PC. The PC two states of membrane voltage, the “up” and “down” state are visible. **(B)** Schematic representation of the two receptor model. In this case the receptor with high affinity (green) is localized to soma and proximal dendrite and the Low affinity receptor (orange) is localized to the axon hillock and initial segment. This compart-mental distribution resembles the distribution of CRF receptors in PCs. **(C)** Schematic plot of the two receptor's affinities (upper panel). Activation of the receptors changes the PCs probability to be in an “up” state. The relationship between the neuromodulator concentration and the probability of the cell to be in an “up” state is depicted in the lower panel. Left of the black dashed line the PC has a probability to be in a “down” state. If the neuromodulators levels are to the right of the black dashed line the PC will be in an “up” state. **(D)** One second puff of 1 μM of CRF (red underline) shifts the Cell to an “up” state. In our toy model it means the concentration of CRF was to the right of the dashed black line. **(E)** Complex spike shifts the PC between the membrane voltage states (upper panel). In the presence of CRF (red underline) the complex spike was unable to shift the cell to a “down” state. The PC became less “sensitive” to input from the olivo- cerebellar loop, due to modulation by CRF.

One of the most characteristic features of PCs is their high firing rate, which can go up to 200 Hz and last for prolonged periods of time (Loewenstein et al., 2005; Shin et al., 2007). It has been proposed that this high firing frequency reflects intrinsic properties rather than the rate of synaptic inputs. Indeed, PC firing, *in vivo* and *in vitro*, persists in the presence of various synaptic blockers. It follows that the intrinsic properties determine the level of firing, upon which the synaptic inputs provides fine modulation. More recently, it was demonstrated that under *in vitro* conditions, as well as under anesthesia, the firing pattern is characterized by abrupt transitions between tonic firing and quiescence (Figure 4A). The terms “up” and “down” states were assigned to denote the firing and the quiescent periods and a corresponding bistable membrane potential has been demonstrated [for a review see (Engbers et al., 2012)]. Whether these transitions occur in awake behaving animals is still debated [see (Yartsev et al., 2009) but (Schonewille et al., 2006)]. Regardless of its outcome, this debate shows the importance of the underlying fact that the firing properties of PCs are robustly modulated between different behavioral states.

### Neuromodulation of Purkinje cells

Table 2 summarizes some of the current knowledge on the neuromodulators operating in the cerebellar cortex particularly on PCs. We then use this case to discuss and compare the possibilities of phasic and tonic effects of neuromodulation.

**Table 2:** **Neuromodulators of Purkinje cells**.

**Neuromodulator**	**Source**	**Receptors**	**Known effects**
5-HT	MdR, PnR And Serotonergic neurons in precerebellar regions (Bishop and Ho, 1985).	5-HT1A (Expression decreases in adults), 5-HT2A,B, 5-HT5A, 5-HT7 (Pazos and Palacios, 1985; Pazos et al., 1985; Kinsey et al., 2001; Geurts et al., 2002).	*In vitro*
			Augmenting the HCN current (Li et al., 1993). Altering the bi-stability of PCs (Williams et al., 2002).
			Increases PC excitability by decreasing the IA current (Wang et al., 1992).
			Decreases PC firing rate through activation of 5-HT1A, Applying 5-HT while blocking 5-HT1A causes an increase in firing rate (Darrow et al., 1990).
			*In vivo*
			Opposes changes in PC firing rate: increasing the rate when it becomes smaller and decreasing it when it becomes higher (Strahlendorf et al., 1984) effect can be species (Kerr and Bishop, 1992) and anesthesia (Strahlendorf et al., 1988) dependent.
			Reduces inward current caused by excitatory input (Hicks et al., 1989).
NE	LC (Watson and McElligott, 1984; Loughlin et al., 1986a,b).	Alpha adrenoreceptors 1A,B (low levels), D(very low levels) (Day et al., 1997), alpha adrenoreceptors2 (Nicholas et al., 1993) and beta adrenoreceptors2 (Wanaka et al., 1989).	*In vitro*
			Increases IPSCs amplitude. Mechanism is both post-synaptic (Woodward et al., 1991) and pre-synaptic (Saitow et al., 2000).
			*In vivo*
			Decreases the firing rate of PCs (Woodward et al., 1991).
Ach	Vestibular nuclei (non-beaded fibers) PTg, GiC and Raphe nuclei (beaded fibers) (Jaarsma et al., 1997).	Musacrenic receptors expression, mainly m2, is seen in the PCs layer in a species dependent fashion (Jaarsma et al., 1995) and Nicotinic receptors (Wada et al., 1989; Graham et al., 2002).	*In vivo*
			Decreases the firing rate of PCs by activation of nicotinic receptors (De La Garza et al., 1987).
Dopamine	VTA (Ikai et al., 1992).	DAT presence is demonstrated (Delis et al., 2004, 2008) and D2,D3,D4,D5 receptors (Khan et al., 1998, 2000; Kim et al., 2009).	*In vitro*
			Autocrine release from PCs. causes a slow inward cation current. (Kim et al., 2009).
Histamine	TMN (Haas and Panula, 2003).	H1, H2, and H3 (Drutel et al., 2001; Takemura et al., 2003).	*In vitro*
			Causes release of calcium from intracellular storages (Kirischuk et al., 1996).
			Increases PC firing rate through activation of H2 (Tian et al., 2000).
Various neuropeptides			Summarized in a review by Ito (2009).

Receptors for the same modulator having different affinity might play a key role in the ability of cerebellar system to respond to tonic and phasic neuromodulatory signals, providing the system with the ability to modulate its processing over different time scales while preserving its ability to response to transient signals. In the tonic release state, where a low level of the modulator is present, the high affinity receptor will be activated (Figure 4C). Thus, it is likely that this receptor will monitor the different basal level of the neuromodulator, providing long time scale modulation. When a sudden increase in neuromodulator occurs, the second receptor will be activated, enabling the system to respond to transient signals. As mentioned above, tonic and phasic release is a common strategy in neuromodulatory systems [reviewed in depth in Dayan (2012)].

The CRF system in the cerebellum is an example of a tonic and phasic modulation system. CRF, a neuropeptide, is released from both the MFs and the CFs (Cummings et al., 1989; Errico and Barmack, 1993). In the cerebellar cortex, the two CRF receptors CRF-R1 and CRF-R2 are expressed in all of the PCs. The two receptor types have a compartment specific distribution pattern (illustrated in Figure 4B) (King and Bishop, 2002; Lee et al., 2004). The properties of the olivo-cerebellar CRF system that support phasic and tonic modulation by the same modulator are: (1) Two receptor types with different affinity to CRF (Lovenberg et al., 1995) (2) A tonic and phasic components [although this hasn't been thoroughly examined, there are some supporting evidence; (Barmack and Young, 1990; Tian and Bishop, 2003; Beitz and Saxon, 2004)] (3) Two distinct sources of CRF, the CF, and MF systems. The first two properties allow the CRF system to be sensitive to tonic and phasic signals and the third provides the possibility of a different functional role to each pathway.

The diverse effects CRF have on PCs are well documented. One study showed that by itself CRF doesn't induce a change in the simple spike firing rate but attenuates the increase in the simple spike firing rate in response to excitatory neurotransmitters (Bishop, 1990). In our ongoing research we demonstrate that in an *in vitro* preparation, application of CRF tends to shift PCs into their firing mode (Libster et al., 2010) (Figure 4D). Other studies demonstrated an increase in the PCs firing rate in response to CRF (Bishop and King, 1992; Bishop, 2002). CFs have a basal firing rate, so we can view the CFs as setting the tonic levels of CRF, and by activation the CRF-R1 (Figure 4B green), increasing the PCs excitability without increasing PCs firing rate. A phasic increase in CRF, either due to increase in the CF activity or released from MFs, will activate the low affinity CRF-R2 receptor which is located mainly on the PCs axon initial segment (Figure 4B orange) (Bishop et al., 2000). We propose, therefore, that cerebellar CRF system is organized in a way that low tonic level, CRF serves to modulate the sensitivity of PCs to excitatory input and in higher level it directly increase PC's firing rate (Figure 4E). Increasing the firing rate lowers the probability of PCs transitions to a down state and reduces the sensitivity to external inputs (Figure 4E). This possible model mechanism is realized in Figure 4C. The sensitivity of the two receptors is depicted as dose response curves in Figure 4C and can be translated into the probability to shift to an up state (Figure 4C). In our hypothetical model the steeper probability curve of the low affinity receptor denotes a threshold like response to CRF level. Activating the high affinity receptors increases the probability to shift to firing mode (Figure 4D). Activating the low affinity receptors directly activates the PCs (orange line, Figure 4C). The entire range of electrical activity is grossly divided into two types of behavior (dashed vertical line). At low concentration of CRF, PC will shift to its firing mode upon synaptic input. At high concentration of CRF PCs shift to their firing mode, where the rate of firing increases with CRF levels. A transient increase in CRF level may, therefore, shift the PC to a continuous firing rendering it more input insensitive.

## The olivary neurons

### The biophysical properties of olivary neurons and network

**Figure 5:**
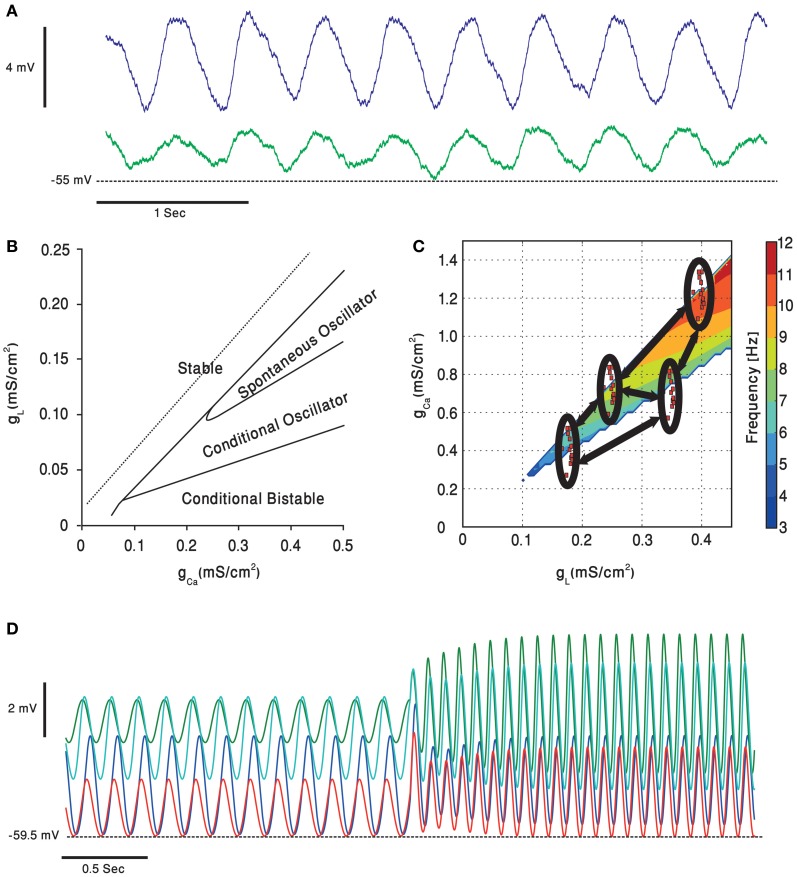
**Subthreshold oscillations of the olivary neurons membrane potential. (A)** Whole cell recording, in an *in vitro* preparation, of two coupled olivary neurons oscillating together. **(B)** A simulated olivary neuron behavior on dependence on the calcium and potassium leak conductance [adapted from Manor et al. (1997)]. Oscillations spontaneously occur in a restricted part of the plane. **(C)** Dependence of the olivary neurons subthreshold oscillations frequency, in the clustered olive model, on potassium leak and calcium conductance [adapted from Torben-Nielsen et al. (2012)]. Each of the clusters (red dots inside black ellipses) contains cells having, roughly, the same value of conductance. The cells inside the clusters have higher coupling coefficients relative to each other and lower coupling strength (marked in black arrows) to cells from other clusters. **(D)** The clustered olive model provides the olivary cells with phase invariance relative to the frequency. Each of the voltage traces is taken from a cell in a different cluster. The cells oscillate in a phase, relative to each other, which is preserved when the oscillation frequency is suddenly increased [adapted from Torben-Nielsen et al. (2012)].

The role of the inferior olive, at least by some researchers, is to endow the cerebellar system with timing capabilities (Xu et al., 2006; Jacobson et al., 2008; Liu et al., 2008; Llinas, 2009). The electrophysiological manifestation of the timing capability is the subthreshold membrane potential oscillations (Figure 5A), reflecting the network organization of the nucleus. It has been suggested that these oscillations emerge when a sufficient number of neurons are electrically coupled (Manor et al., 1997; Devor and Yarom, 2002; Torben-Nielsen et al., 2012) Indeed, electrical coupling between olivary neurons was demonstrated in physiological experiments and gap junction, the structural correlate of electrical connection, was identified in ultrastructure examinations (Llinas et al., 1974; Sotelo et al., 1974; Devor and Yarom, 2002; Leznik and Llinas, 2005). The gap junctions are located between dendritic spines and surrounded by inhibitory and excitatory synaptic terminals, forming a distinct structure known as the olivary glumerulus (King, 1976; De Zeeuw et al., 1998). This special arrangement indicates that the coupling strength is under synaptic regulation and therefore the formation of an oscillating network is controlled by synaptic inputs (Devor et al., 2001; Bazzigaluppi et al., 2012). The inhibitory input to the olivary glumerulus is provided by the inhibitory projection neurons of the deep CN (Bazzigaluppi et al., 2012) that are directly controlled by the PC's inhibitory input. The output of the olivary neurons ascends to the cerebellar cortex where it forms the CF synapse, thereby completing the olivo-cerebellar loop (Uusisaari and De Schutter, 2011).

Several models have been proposed to account for network dependent subthreshold voltage oscillations. The heterogeneity model assumes that olivary neurons are a heterogeneous population of neurons that differ in the density of their calcium and leak channels. This heterogeneity results in different types of neuronal behavior (see Figure 5B) that upon coupling generate subthreshold oscillations. In a recent study it was demonstrated that coupling strength can also shift the frequency of oscillations (Figure 5C), provided that the network is organized in clusters of coupled neurons (Torben-Nielsen et al., 2012). It follows that any global effect on calcium or leak channels, like that provided by neuromodulators, can alter the oscillation's frequency and thereby modulates the timing signal generated by the olivo-cerebellar loop.

### Neuromodulation of olivary neurons and network

Table 3 summarizes some of the current knowledge on the neuromodulators operating within the inferior olive nucleus. Our consideration on the functional effects of neuromodulators on olivary activity is based on two assumptions. First, the subthreshold oscillations are the source for cerebellar time coding and time representation. This timing capability serves both sensory and motor function. In sensory processing, time serves to predict motor outcomes, whereas in motor processing it provides temporal patterns to accurately execute motor commands. Second, we assume that the subthreshold oscillations are generated from complex interactions between intrinsic membrane properties and electrotonic connections that are best described by our heterogeneity model (see above).

**Table 3:** **Neuromodulators of the inferior olive**.

**Neuromodulator**	**Source**	**Receptors**	**Known effects**
5-HT	MAO receives from ROb and RPa and DAO from GiC (Wiklund et al., 1981a; Bishop and Ho, 1986). Release sites can be either junctional (close to the synapses) or non junctional (Wiklund et al., 1981b).	5-HT2A, 5-HT5B (Kinsey et al., 2001).	*In vitro*
			Facilitates HCN current, reduces the inward rectifying potassium current and LVA calcium current (Placantonakis et al., 2000).
			*In vivo*
			Increases the average firing rate of inferior olivary neurons and slowing their oscillation frequency (Sugihara et al., 1995).
NE	LC (Kobayashi et al., 1974).	α-adrenoreceptors1A,B (both with low levels of expression) and D (high levels of expression Day et al., 1997). α-adrenoreceptors2 (Probst et al., 1984) and β-adrenoreceptors2 (Wanaka et al., 1989).	
Ach	Species dependent, i.e., in cat ChAT-immunoreactive fibers were found in the entire IO (Kimura et al., 1981) while in other species only the dorsal cap of the MAO was innervated by fibers projecting from nucleus prepositus hypoglossi and the medial aspect of the medial vestibular nuclei.	Nicotinic (Swanson et al., 1987; Wada et al., 1989) and muscarinic (Wamsley et al., 1981) receptors.	
Dopamine	Prerubral parafascicular area (mainly to the ventrolateral outgrowth) (Toonen et al., 1998).	D2 and D3 receptors (Bouthenet et al., 1987, 1991).	
Histamine	TMN (Inagaki et al., 1988; Haas and Panula, 2003).	H1, H2 and H3 (low levels) (Schwartz et al., 1991).	

With these assumptions one should wonder: should representation of time be modulated? Do we need a different representation of time in different behavioral sates? It is rather difficult, if not impossible, to answer these questions. It seems inevitable to conclude that time representation should be accurately preserved irrespective of the behavioral state. After all keeping accurate time is crucial for survival. Keeping accurate time for predicting motor outcome is definitely essential, but execution of motor commands is, and should be, modified upon a shift in the behavioral state. Motor performance is affected by the current level of alertness and motivation. Increase alertness is associated with faster movement time (Gray, 2011; Shiner et al., 2012). Therefore, an increase in movement velocity that maintains the temporal structure of the movement entails a change in the temporal pattern generated by the cerebellar system. We propose that these contradictory needs, preserving time representation for sensory function and altering timing of motor execution, are manifested in the non-homogeneous innervations of the olivary complex by neuromodulators. In the case of serotonergic innervations, some parts are heavily innervated while others are almost or completely devoid of such innervations (Wiklund et al., 1977; Leger et al., 2001). Thus, if behavioral state defines the serotonin level, the function will be modified in some olivary subnuclei while preserved in others.

To understand how temporal patterns can be modified in a way that will support faster movements one should consider the heterogeneity model. The gl-gCa plane shown in Figure 5C demonstrates that the higher the leak and the calcium conductance, the higher is the frequency of oscillation. Serotonin decreases both conductance and therefore a lower frequency is expected and indeed was experimentally observed (Sugihara et al., 1995; Placantonakis et al., 2000). We previously demonstrated that under harmaline intoxication sudden shifts in frequency of cortical complex spikes activity was frequently observed (Jacobson et al., 2009; Choi et al., 2010). Interestingly during this frequency shift, the phase difference between neurons was maintained. Similar phenomenon is also predicted by our heterogeneity model (Figure 5D). It is tempting to suggest that neuromodulators can change the frequency of the temporal pattern while maintaining the temporal order within the pattern, thus producing faster movement while maintaining coordination.

## Concluding remarks

This short review is focused on the effects of modulatory agents that operate within the olivo-cerebellar system. Beyond references to published studies, we presented various hypothetical possibilities by which neuromodulators can exert differential effects that are both spatial and temporal specific. We speculate that such mechanisms endowed the system with the capabilities to adjust cerebellar processing to a given behavioral state.

### Conflict of interest statement

The authors declare that the research was conducted in the absence of any commercial or financial relationships that could be construed as a potential conflict of interest.

## Abbreviations

bPN: Big Projection Neuron
CF: Climbing Fibers
CN: Cerebellar Nuclei
DAO: Dorsal Accessory Olivary Nucleus
DN: Dentate Nucleous
DRN: Dorsal Reticular Nucleus
GiC: Nucleus Reticularis Gigantocellularis/Paragigantocellularis Complex
IN: Interposed Cerebellar Nucleus
IO: Inferior Olive
LC: Locus Coeruleus
MAO: Medial Accessory Olivary Nucleus
MdR: Medullary Reticular Formation
MF: Mossy Fibers
PC: Purkinje Cell
PeFLH: Perifornical Part of Lateral Hypothalamus
PnO: Oral Pontine Reticularis nucleus
PnR: Pontine Reticular Formation
PTn: Pedunculopontine Tegmental Nucleus
ROb: Raphe Obscurus Nucleus
RPa: Raphe Pallidus Nucleus
TMN: Tuberomammillary Nucleus
VTA: Ventral Tegmental Area.
